# Fabrication of paper-based enzyme immobilized microarray by 3D-printing technique for screening α-glucosidase inhibitors in mulberry leaves and lotus leaves

**DOI:** 10.1186/s13020-019-0236-y

**Published:** 2019-03-29

**Authors:** Shangxin Guo, Xiaotong Lin, Yi Wang, Xingchu Gong

**Affiliations:** Pharmaceutical Informatics Institute, College of Pharmaceutical Sciences, Zhejiang University, Hangzhou, 310058 China

**Keywords:** Paper-based microarray, Ligand fishing, α-Glucosidase, 3D printing

## Abstract

**Background:**

The discovery of bioactive compounds in traditional Chinese medicine (TCM) has become an important field in TCM modernization. Ligand fishing is a suitable method for discovery of bioactive compounds in complex mixtures such as TCM with high selectivity. Because of unique advantage of low cost and convenience, paper-based microdevices can be good carriers for enzyme immobilized ligand fishing.

**Methods:**

As an important enzyme for glucose metabolism, α-glucosidase was immobilized on polycaprolactone–chitosan-modified paper to prepare the microdevice with unique microfluid structure generated by 3D printing technology, which can be easily applied to screen active compounds in herbal extracts. The preparation conditions of the paper microarray were optimized. The activity of immobilized α-glucosidase was verified by colorimetric reactions which can be easily monitored by cellphone. The paper microarray with α-glucosidase immobilized was used to screen active compounds in the water extracts of mulberry leaves and lotus leaves.

**Results:**

Several key parameters including Na_2_CO_3_ solution concentration, Na_2_CO_3_ solution volume, glutaraldehyde concentration, crosslinking time of glutaraldehyde and time of α-glucosidase immobilization were optimized. The proposed paper-based microarray was successfully applied in screening active compounds in two herbal extracts. Four compounds including chlorogenic acid, quercetin-3-*O*-glucuronide, isoquercetin, and quercetin were identified as α-glucosidase inhibitors. The compounds with significant non-specific adsorption caused by chitosan, such as isoquercitrin, astragalin, quercetin, were also found to be active compounds.

**Conclusions:**

An enzyme immobilized paper microarray was designed and fabricated in this work. Polycaprolactone and chitosan were used to modify filter paper to prepare paper microarrays. Parameters of paper device preparation were optimized. Our findings suggested that 3D-printing paper-based microarrays can be a simple and low-cost approach for discovery of active compounds of TCM.

**Electronic supplementary material:**

The online version of this article (10.1186/s13020-019-0236-y) contains supplementary material, which is available to authorized users.

## Background

Novel omics techniques including transcriptomics, proteomics, metabolomics as well as microarrays have greatly contributed to the modernization of traditional Chinese medicine (TCM) in chemical and pharmacological researches. Microarray assays have been widely applied in screening and characterization of TCM [1]. Microarrays can be used to identify bioactive components from TCM and target biologically active molecules in TCM [2]. Zhang et al. [3] incorporated the ITS1-5.8S-ITS2 sequences of 16 *Dendrobium* species on a glass slide to fabricate a DNA microarray. It was used to detect the presence of D. nobile in a Chinese medicinal formulation containing nine herbal components. Wang et al. [4] identified the potential targets of the ingredients of Xuesaitong by integrating microarray data, text mining and pharmacophore model-based prediction. Notoginsenoside R_1_, ginsenoside Rg_1_, Rb_1_, Rd and Re were found to be the major bioactive compound and were validated. Bioactive compound discovery is an important part of the TCM modernization [5]. The discovered active compounds can be used as the quality markers of the TCM [6]. They can also be used for developing new drugs. However, the discovery of bioactive compounds in TCM is usually difficult because of the very complex composition of TCM systems. In most cases, the compounds in TCM systems are extracted with different solvents, and then separated by preparative chromatography. After that, chemical structure and biological activity will be further determined. However, it is time-consuming and labor-intensive. Thus, it’s urgent to develop effective methods for the discovery of bioactive compounds in TCM.

Ligand fishing is a technical method to separate bioactive compounds from complex mixtures with high selectivity. The method is achieved by interacting receptors from ligands on the basis of intermolecular affinity [7–9]. Ligand fishing can be carried out with or without the ligand immobilized on a carrier. The captured receptors can be separated by washing and detected by mass spectrometry or other detectors. Due to high selectivity of the ligand fishing method, it’s possible to obtain bioactive compounds for a specific target from complex systems directly. At present, ligand fishing is widely applied to separate active compounds from complex products including plant extracts and cell extracts [10]. It has been widely reported that magnetic beads, hollow fiber membranes, etc. were applied as carries for ligand fishing to discover active compounds from the TCM. Liu et al. [11] prepared nano-magnetic beads bonding with bovine serum albumin and used chromatography-mass spectrometry to screen active ingredients in the Pueraria lobate. Thirteen active ingredients were screened. Deng et al. [12] performed a new screening assay based on ligand fishing using magnetic Fe_3_O_4_@SiO_2_-COX-2 combined with high-performance liquid chromatography-diode array detector-mass spectrometry to screen and identify COX-2 inhibitors from green tea. It was a simple, robust and reproducible approach to discover COX-2 inhibitors from complex matrix. Tao [5] established a hollow fiber adsorption screening method for triglyceride inhibitors to discover active compounds in lotus leaf extract. Three active compounds were found having inhibitory activity on triglyceride. Overall, ligand fishing is quicker, more effective, and more sensitive for discovery of active compounds in TCM compared with traditional methods [13].

The paper device, a kind of analytical device made of paper, is developed rapidly in recent years. Since Martinez et al. [14] introduced paper as microfluidic device, paper devices have been widely used as analytical devices in chemical reactions and environmental monitoring. It showed that it’s low-cost, easy to use, and capable of multiplexed chemical analysis [15–18]. The paper devices own high specific surface area which makes it easy to combine molecules to adsorb proteins. Used paper devices can be handled easily through burning them, reducing pollution caused by experimental consumables. White paper provided ideal background signals for observing colorimetric reactions. Kaewarse et al. [19] performed a reduction reaction of nitro blue tetrazolium on the paper-based chip to detect enzymatic activity and the results of the reaction showed purple on the paper. Qualitative and quantitative results can be obtained combining some portable detectors such as cellphones and cameras. Therefore, it is possible to judge the immobilization effects of enzymes on paper easily. There are also some examples of paper devices with commercial successes, such as pregnancy test strips and pH test paper.

In this work, an effective omics technology based on paper device was proposed. Paper-based microarrays were prepared and applied to discover active compounds in TCM extracts. Polycaprolactone–chitosan-modified paper was prepared with the assistance of 3D printing technology. α-Glucosidase was immobilized on the modified paper. The preparation conditions of paper microarrays were optimized. α-Glucosidase was one of the targets of diabetes. It’s reported that mulberry leaves and lotus leaves are effective on treatment of hyperglycemia in type 2 diabetes [20, 21]. Therefore, the paper microarray was then used to discover active compounds from mulberry leaf extracts and lotus leaf extracts in this work.

## Methods

The Minimum Standards of Reporting Checklist contains details of the experimental design, and statistics, and resources used in this study (Additional file 1).

### Materials and chemicals

Ethanol (≥ 99.7%) and glacial acetic acid (≥ 99.5%) were purchased from Shanghai Lingfeng Chemical Reagent Co., Ltd. (Shanghai, China). Chitosan and α-glucosidase were purchased from Merck (Darmstadt, Germany). Glutaraldehyde solution (50%) was purchased from Sangon Biotech Co., Ltd. (Shanghai, China). 4-Nitrophenyl-α-d-glucopyranoside (PNPG) was purchased from Shanghai Aladdin Bio-Chem Technology Co., Ltd. (Shanghai, China). Sodium chloride (≥ 99.5%), disodium hydrogen phosphate dodecahydrate (≥ 99.0%), potassium chloride (≥ 99.5%), potassium dihydrogen phosphate (≥ 99.5%) and sodium carbonate (≥ 99.8%) were purchased from Sinopharm Chemical Reagent Co., Ltd. (Shanghai, China). Mulberry leaves and lotus leaves were purchased from Huadong Herbal Slice Co., Ltd. (Hangzhou, China). Polycaprolactone was purchased from Dongguan Top Cool Electronics Technology Co., Ltd. (Dongguan, China). Quantitative filter paper was purchased from Hangzhou Wohua Filter Paper Co., Ltd. (Hangzhou, China). Ultrahigh-purity water was produced using a water purification system (Milli-Q, Millipore, US).

### Preparation of filter paper immobilized with α-glucosidase

**Fig. 1:**
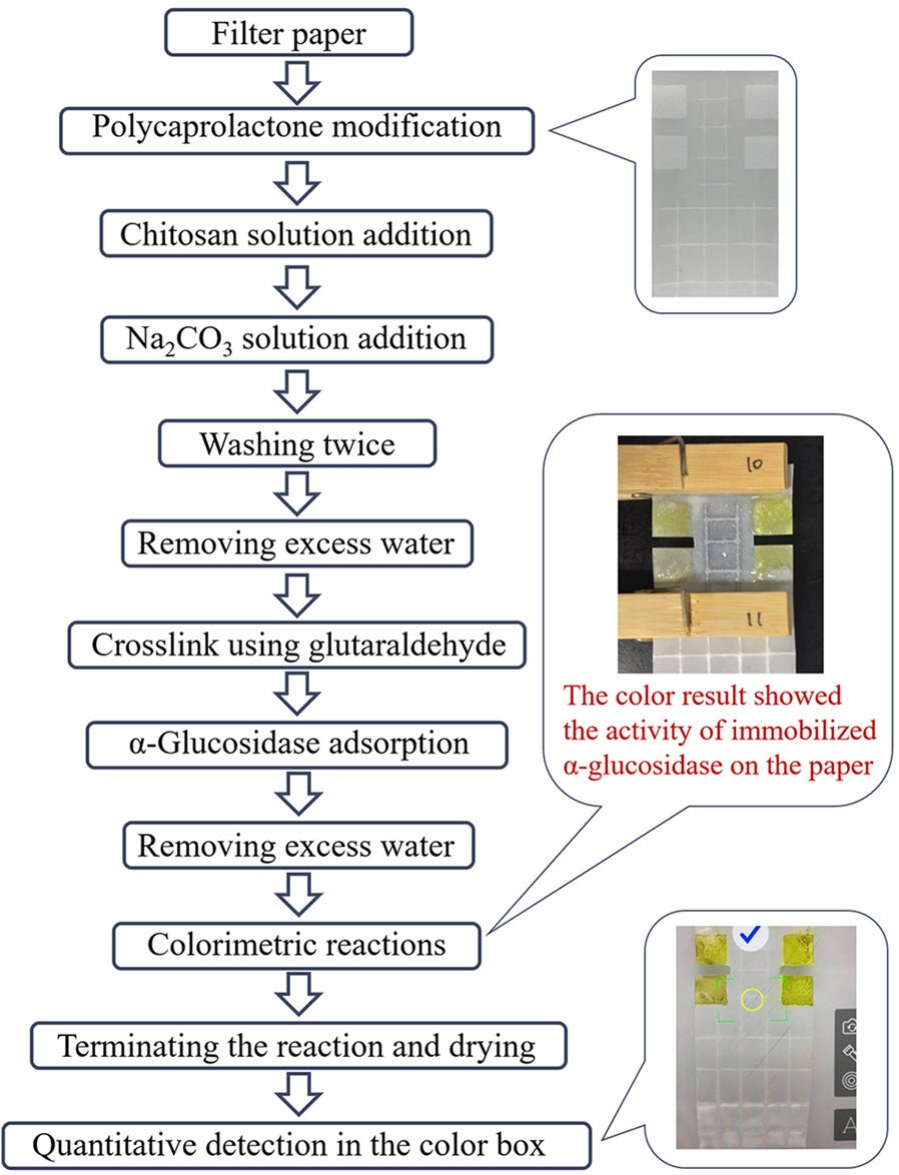
The process of preparation of filter paper immobilized with α-glucosidase

In this work, filter paper was firstly modified with 3D printed polycaprolactone to form hydrophobic zones. After that, the filter paper was cut carefully to get a modified paper with both hydrophilic and hydrophobic zones. Chitosan was then used to modify the hydrophilic zone. After that, α-glucosidase was immobilized on the hydrophilic zone. The activity of α-glucosidase was tested by a colorimetric reaction. The results were collected by a cellphone (P9, Huawei Technologies Co., Ltd. China). Figure 1 shows the above-mentioned processes.

#### Polycaprolactone-modification of filter paper

**Fig. 2:**
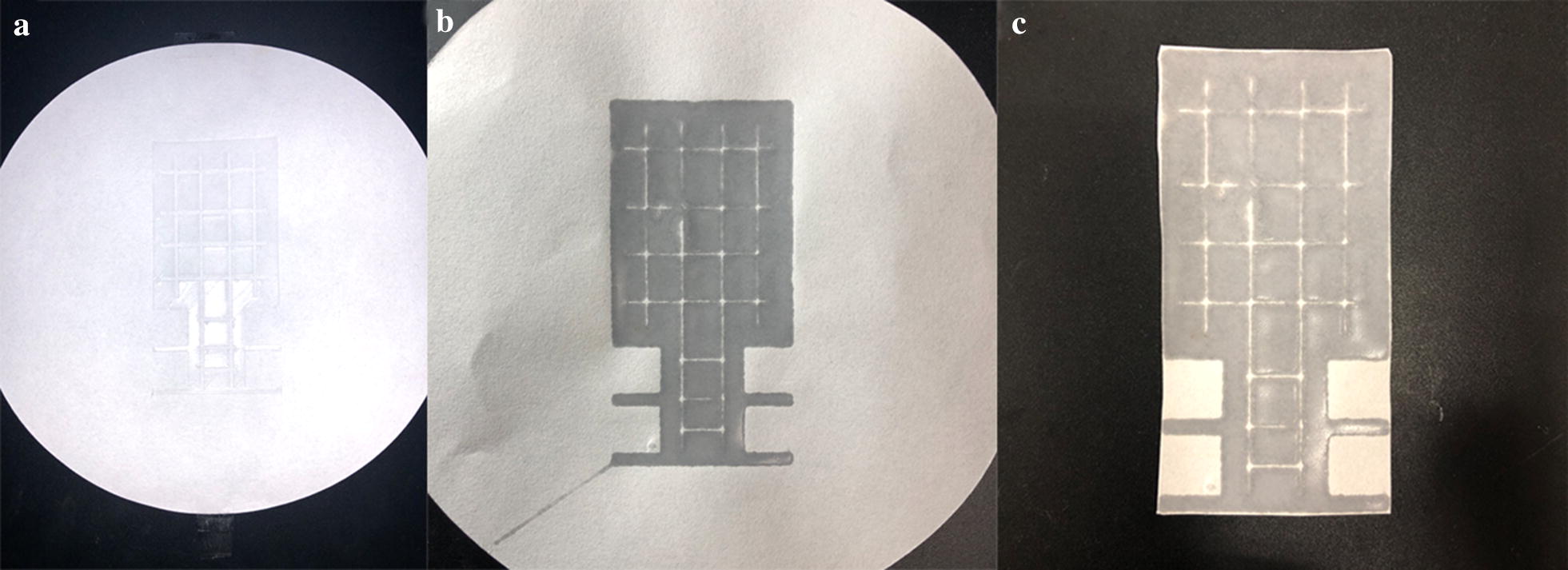
Polycaprolactone-modification of filter paper: **a** polycaprolactone 3D-printed on filter paper; **b** the polycaprolactone-modified paper; **c** the polycaprolactone-modified paper after cutting

The pattern of paper microarrays was designed using a drawing software (CAD2014, Autodesk Inc.) and printed on the filter paper by a 3D printer (Einstart-S, Hangzhou Shining 3D Co., Ltd. China). A piece of filter paper was stuck on the 3D printing platform and polycaprolactone was printed on it, as seen in Fig. 2a. Then the filter paper with polycaprolactone on it was heated in an electric baking pan (JK-3030S2, Joyoung Co., ltd. China) at 150 °C for 2 h. Then the filter paper was taken out the pan and cooled in ambient temperature for at least 5 min. After that, polycaprolactone-modified paper was cut with scissors, as seen in Fig. 2b. Hydrophilic regions consisted of 4 squares with side length of about 10 mm. The polycaprolactone-modified paper was shown in Fig. 2c.

#### Chitosan-modification of filter paper

Chitosan is reported to be a good material to immobilize enzyme and retrain enzyme activity [22, 23]. A solution of chitosan (2.5%, m v^−1^) was prepared in acetic acid (2.5%, v v^−1^). A certain amount of chitosan solution was pipetted into the hydrophilic region and dried in ambient temperature for at least 1 h. Na_2_CO_3_ solution was added to the hydrophilic region to remove excess acetic acid. To remove excess Na_2_CO_3_, the region was then immersed in ultrahigh-purity water for 5 min under a magnetic stirring of 200 rpm twice. After that, the polycaprolactone–chitosan-modified paper was dried in ambient temperature for at least 30 min.

#### Immobilization of α-glucosidase on the polycaprolactone–chitosan-modified paper

Glutaraldehyde possesses the ability to react with multiple groups in protein molecules, such as amino group. Therefore, glutaraldehyde was selected as the crosslinker. 0.18 mol/L glutaraldehyde solution (50 μL) was pipetted into the hydrophilic region to crosslink chitosan on paper for a period of time. Phosphate buffered saline (PBS, pH = 7.2) was prepared for the preparation of 5 U/mL α-glucosidase solution and 2.5 mM PNPG solution. α-Glucosidase solution (50 μL) was added to 3 hydrophilic square regions respectively for a period of immobilization. These 3 regions would be used as triple repetition. The 1 region left was added with PBS solution (50 μL) as the blank control. Then hydrophilic regions with α-glucosidase immobilized were washed in ultrahigh-purity water under magnetic stirring with a speed of 200 rpm for 5 min twice. The paper microarray was dried in ambient temperature for at least 30 min after washing. PBS solution (20 μL) and PNPG solution (50 μL) were added to the hydrophilic region for reaction. 30 min later, 0.5 M Na_2_CO_3_ solution (50 μL) was added to the reaction region to put an end to the reaction. Finally, the paper device was dried in a drying oven (DHG-9123A, Shanghai Jinghong Experimental Equipment Co., Ltd. China) at 50 °C for at least 10 min.

#### Color data collection from dried paper microarrays

**Fig. 3:**
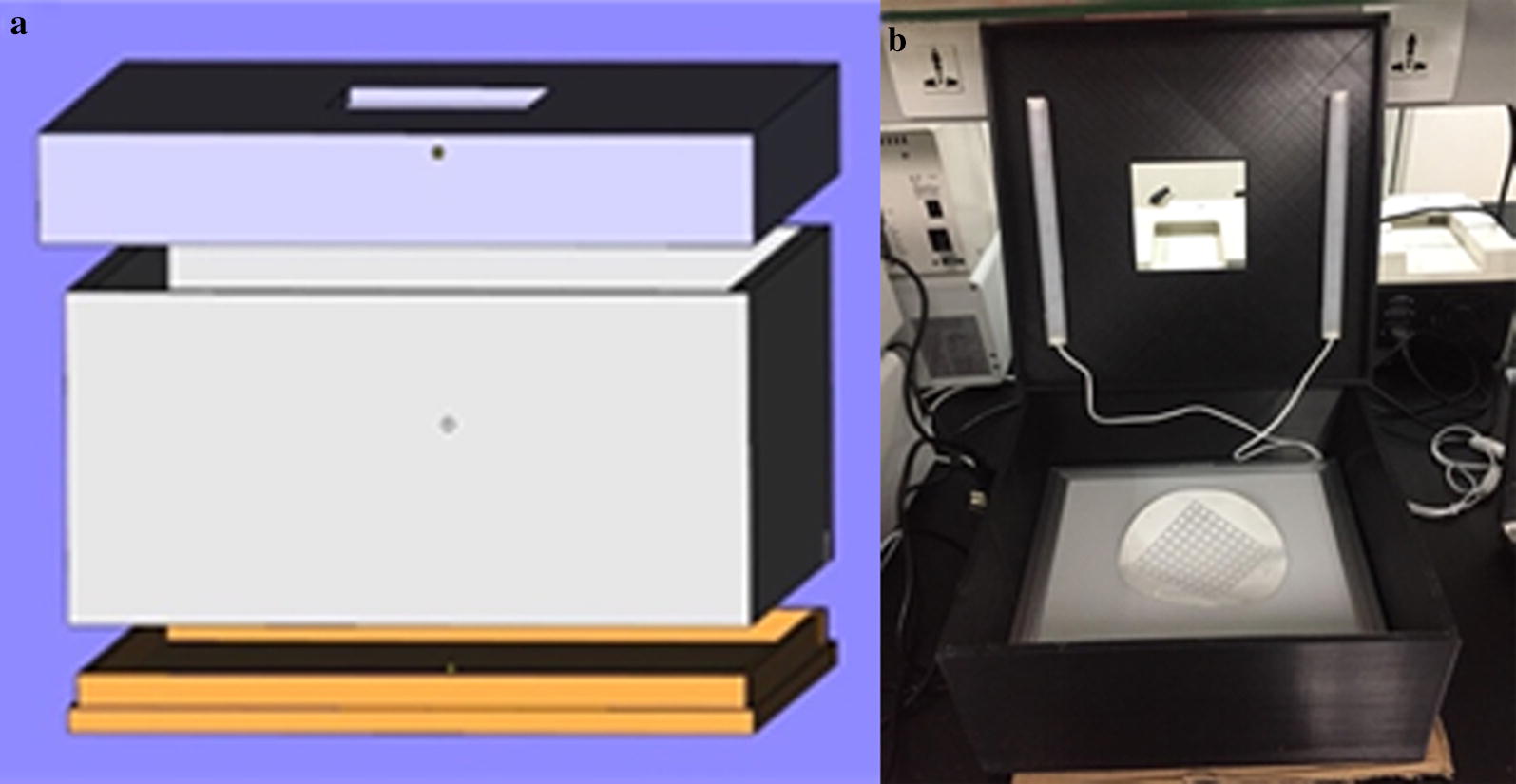
The box made using 3D printing technology with fixed light sources: **a** the formation of the box; **b** the photo of the box interior

The dried paper microarray was placed in a box prepared through 3D printing with fixed light sources as seen in Fig. 3. A cellphone was used to read color data from paper microarrays placed in the box. The color data was read with an app (Color grab, Loomatix Ltd.). In previous work [24], the color component Y of CMYK color mode was selected to measure colorimetric reaction results to characterize the activity of α-glucosidase. Higher Y value indicates higher α-glucosidase activity after immobilization. The average Y value obtained from 3 regions with α-glucosidase immobilized was subtracted from the Y value obtained from the region with no α-glucosidase to obtain a corrected value as the response.

### Optimization of enzyme immobilization

In order to improve α-glucosidase activity after immobilization, optimization of conditions for the polycaprolactone–chitosan-modified paper was carried out. Six parameters including the chitosan solution volume, the Na_2_CO_3_ volume, the Na_2_CO_3_ concentration, the glutaraldehyde concentration, the time of glutaraldehyde crosslinking with chitosan, and the time of α-glucosidase crosslinking with the paper microarray were optimized using one-factor-at-a-time method. There is triple repletion in each experiment. In the optimization, higher Y value was favored.

### Detection of active compounds in mulberry leaves and lotus leaves

#### Preparation of the mulberry leave extract and the lotus leave extract

Mulberry leaves (50.0 g) were immersed in ultrahigh-purity water (600 mL) for 30 min. Then the mulberry leaves were extracted at reflux for 30 min. The extract was obtained by filtering. Ultrahigh-purity water (500 mL) was supplied for the second reflux extraction. Mulberry leaves were extracted at reflux for 20 min. The extract was obtained again by filtering. Two aqueous extracts were mixed and concentrated at 60 °C at reduced pressure. 250 mL of concentrate with a concentration of 0.2 g material/mL was collected. Then the concentrate was stored in a refrigerator (BL-240/241L, Shanghai Yisi Technology Industrial Co., Ltd. China). The lotus leaf extract was prepared in the same way as the mulberry leaf extract.

#### Detection of active compounds

The α-glucosidase immobilized paper was prepared at optimized conditions. Before ligand fishing, the α-glucosidase immobilized paper was washed in ultrahigh-purity water using a magnetic stirring with a speed of 200 rpm for 5 min twice. Then the paper microarray was immersed in the extract of mulberry leaves or lotus leaves under stirring with a speed of 200 rpm for 60 min. After that, active compounds were expected to be adsorbed on the paper microarray. The paper microarray was then washed using the magnetic stirring with a speed of 200 rpm for 5 min twice. The paper microarray was cut into four parts. The separated part was immersed in acetonitrile (2 mL) for 15 min to desorb the active compounds with the assistance of ultrasonic wave (SK1200H, Shanghai Kudos ultrasonic instrument Co., Ltd. China). The eluent was collected, centrifuged, and then analyzed using the high resolution electrospray ionization mass spectroscopy (HREIMS). Mass spectrometric detection was performed using a quadrupole-TOF mass spectroscopy (TripleTOF 5600+, ABSciex, Canada). According to the design of paper microarrays, the acetonitrile sample of the blank control was also obtained.

#### Liquid chromatography–mass spectrometry conditions

The analysis method of mulberry leaf eluent was reported by Zhao et al. [25]. The separation was performed on an Agilent Eclipse XDB C18 column (4.6 mm × 250 mm, 5 μm). The chromatographic conditions were as follows: mobile phase: acetonitrile (phase A) and 0.1% formic acid–water (phase B); linear gradient elution (0–5 min, 9% A; 5–13 min, 9–13% A; 13–22 min, 13–22% A; 22–31 min, 22–34% A; 31–44 min, 34–48% A; 44–54 min, 48–53% A; 54–59 min, 53–65% A; 59–75 min, 65% A; 75–80 min, 65–79% A); flow rate: 1.0 mL/min; column temperature: 30 °C; detection wavelength: 254 nm; injection volume: 10 μL. The mass spectrometry conditions for mulberry leaf samples were as follows: ion source: electrospray ion source; negative scan mode; capillary voltage: 1.8 kV; cone voltage: 40 V; ion source temperature: 120 °C; solvent removal temperature: 500 °C; solvent removal nitrogen flow rate 700 L/h; cone gas volume: 50 L/h; m/z: 100–1200.

The analysis method of lotus leaf eluent was reported by Cheng and Wang [26]. The separation was carried out on an Agilent Eclipse XDB C18 column (4.6 mm × 250 mm, 5 μm). The chromatographic conditions were as follows: mobile phase: 0.1% formic acid–water (phase A) and acetonitrile (phase B); linear gradient elution (0–60 min, 10% B; 60–80 min, 50% B; 80 min, 100% B); flow rate: 0.5 mL/min; column temperature: 30 °C; detection wavelength: 254 nm; injection volume: 20 μL. The mass spectrometry conditions for lotus leaf samples were as follows: ion source: electrospray ion source; negative scan mode; mass range for scan mode: m/z: 100–2000; collision gas: helium gas; electrospray voltage: 4.5 kV; sheath gas flow velocity: 30 arb; auxiliary gas flow velocity: 10 arb; capillary voltage: 15 V.

## Results

### Optimization of the preparation parameters of paper microarrays

Optimization experiments were carried out with the one-factor-at-a-time method. Results of colorimetric reactions on paper microarrays were characterized by corrected Y value. According to the results in previous work [24], higher Y value indicates better immobilization of α-glucosidase.

#### The influence of chitosan solution volume

Chitosan solution of 100, 200, 300, and 400 μL was added to the hydrophilic region of polycaprolactone-modified paper. After adding glutaraldehyde solution, overflow was observed in the hydrophilic regions with 200, 300, and 400 μL of chitosan solution added. Because of the small area of hydrophilic region, it is difficult to load large volume of aqueous solution. Therefore, 100 μL of chitosan solution was used in following research works.

#### The influence of Na_2_CO_3_ solution concentration and volume

**Fig. 4:**
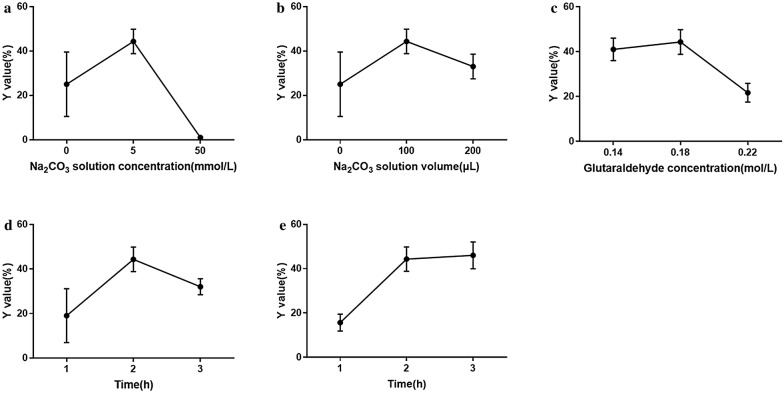
The potential influences on enzyme immobilization: **a** the influence of Na_2_CO_3_ solution concentration; **b** the influence of Na_2_CO_3_ solution volume; **c** the influence of glutaraldehyde concentration; **d** the influence of crosslinking time of glutaraldehyde; **e** the influence of time of α-glucosidase immobilization

Other parameters were fixed and listed in Additional file 2: Table S1. Without adding Na_2_CO_3_ solution, the average Y value was 25% after color reaction, as seen in Fig. 4a. Then 100 μL of Na_2_CO_3_ solutions of different concentrations (5 mM and 50 mM) were added to the paper microarray separately to observe the effects on immobilized α-glucosidase. After reaction, pH values of mixed solution on the paper microarray were measured using test paper. Colorimetric results were also obtained after drying in the oven. The group with 5 mM Na_2_CO_3_ solution added showed neutral and the average Y value was 44% as seen in Fig. 4a. The pH value of the group with 50 mM Na_2_CO_3_ solution added was approximately 8.0 and no colorimetric change appeared on the paper microarray. It seemed that high Na_2_CO_3_ solution concentration resulted in high pH value, which made α-glucosidase inactive. Therefore, optimized Na_2_CO_3_ solution concentration was 5 mM. Different volume of Na_2_CO_3_ solution was compared, as seen in Fig. 4b. The average Y value of 200 μL of Na_2_CO_3_ solution was a little lower than that of 100 μL. Therefore, 100 μL of Na_2_CO_3_ solution was used in following works.

#### The influence of glutaraldehyde concentration

In order to immobilize α-glucosidase firmly on the paper microarray, the chemical crosslinking method was used. Glutaraldehyde was selected as the crosslinker. Glutaraldehyde concentration was optimized in this work. Glutaraldehyde solutions (0.14, 0.18 and 0.22 mol/L) were prepared to test the influence on enzymatic immobilization. Other parameters were fixed and listed in Additional file 2: Table S2. It can be seen in Fig. 4c that the average Y value of the group with 0.18 mol/L glutaraldehyde solution was the highest. Thus 0.18 mol/L of glutaraldehyde solution was selected.

#### The influence of time of glutaraldehyde crosslinking

A period of time is required for the reactions between aldehyde groups of glutaraldehyde and amino groups of chitosan. The effect of crosslinking time between glutaraldehyde and chitosan was compared for 1–3 h. Other parameters were fixed and listed in Additional file 2: Table S3. As seen in Fig. 4d, the largest average Y value was obtained at 2 h crosslinking. Therefore, 2 h of crosslinking time between glutaraldehyde and chitosan was applied for the following works.

#### The influence of time of α-glucosidase immobilization

α-Glucosidase cannot be fully immobilized on the paper microarray if immobilization time is not enough. Therefore, the influence of time of α-glucosidase immobilization was optimized with other parameters fixed and listed in Additional file 2: Table S4. The results can be seen in Fig. 4e. The average Y value became higher with the increase of time. Finally, 3 h of α-glucosidase immobilization was selected to prepare the paper microarray for active compound discovery.

### Active compound detection

#### Mulberry leaves

**Fig. 5:**
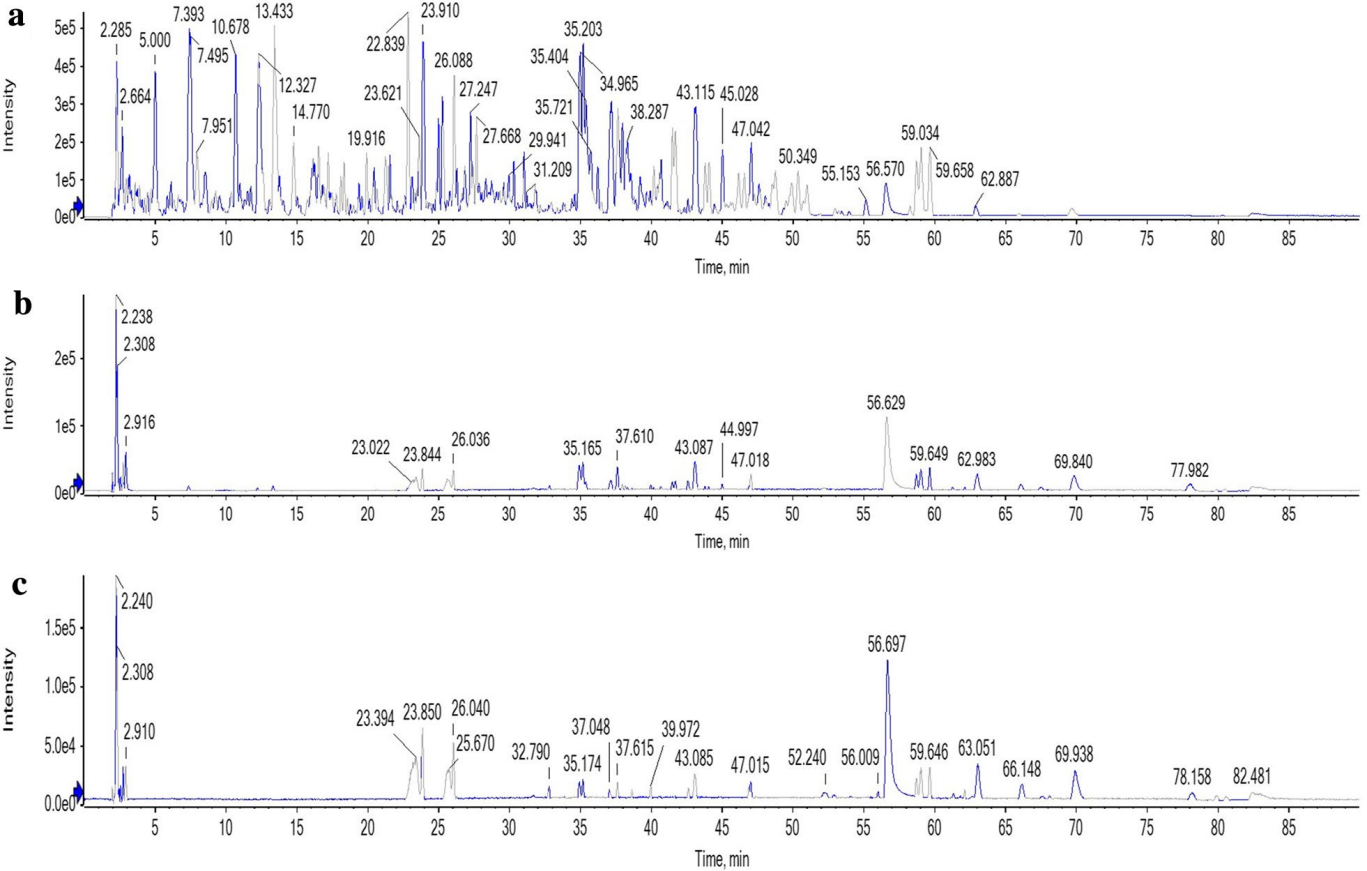
The mass spectrometry chromatogram of total ion current of the mulberry leaf: **a** the mulberry leaf extracts; **b** the eluent of the positive group; **c** the eluent of the blank control

The HREIMS results of the mulberry leaf extract eluent through the paper microarray method are shown in Fig. 5. Peak numbers of eluent samples were much fewer than those of the mulberry leaf extract. According to the retention time of the chromatographic peaks and the information obtained using HREIMS, 4 chromatographic peaks of related substances were identified, which were chlorogenic acid, isoquercetin, astragalin and quercetin. Their information is listed in Table 1. The relative molecular mass accuracy of chlorogenic acid, astragalin and quercetin was less than 5 ppm. The area ratios between peaks of the positive group and peaks of the blank control (A_1_/A_2_) are listed in Table 1. The chlorogenic acid peak area in positive group samples was much higher than that in the blank control sample, which indicated that chlorogenic acid in the mulberry leave extract was specifically adsorbed by the immobilized α-glucosidase. Therefore, chlorogenic acid may be the ligand of α-glucosidase. As reported in Liu et al.’s paper [27], chlorogenic acid possesses the ability to inhibit α-glucosidase activity. Therefore, it was confirmed that active compounds in mulberry leaves included chlorogenic acid.

**Table 1:** The data of compounds in mulberry leaves obtained using HREIMS (A_1_: the peak area of the positive group; A_2_: the peak area of the blank control)

Peak no.	t_R_/min	Identification	Molecular formula	[M−H]^−^			A_1_/A_2_ (%)
				Detected	Expected	Error/ppm	
1	7.475	Chlorogenic acid	C_16_H_18_O_9_	353.0885	353.0873	3.4	248
2	23.844	Isoquercitrin	C_21_H_20_O_12_	463.0840	463.0877	− 8.0	57
3	26.036	Astragalin	C_21_H_20_O_11_	447.0936	447.0927	2.0	66
4	32.826	Quercetin	C_15_H_10_O_7_	301.0360	301.0349	3.7	89

#### Lotus leaves

**Fig. 6:**
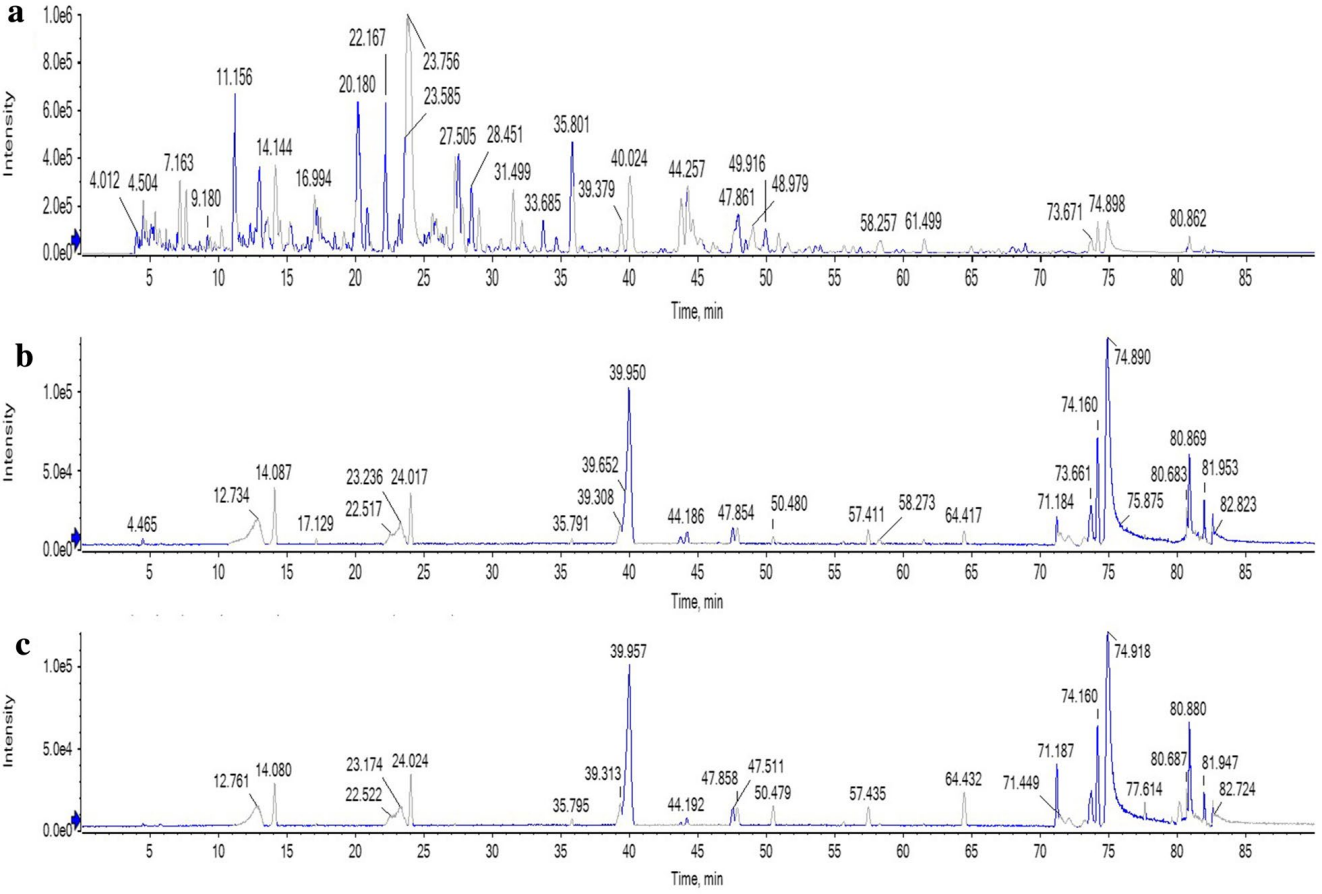
The mass spectrometry chromatogram of total ion current of the lotus leaf: **a** the lotus leaf extract; **b** the eluent of the positive group; **c** the eluent of the blank control

The mass spectrometry chromatogram of total ion current of the lotus leaf extract and the samples prepared with the paper microarray are shown in Fig. 6. Peak numbers of eluent samples were much fewer than those of the lotus leaf extract. According to the retention time of the chromatographic peaks and the mass spectrometry information obtained using HREIMS, 4 chromatographic peaks were identified, which were quercetin-3-*O*-glucuronide, isoquercetin, quercetin, and kaempferol. The information obtained can be seen in Table 2. The relative molecular mass accuracy of 4 compounds was less than 5 ppm. The area ratios between peaks of the positive group and peaks of the blank control (A_1_/A_2_) were listed in Table 2. The peak area of quercetin-3-*O*-glucuronide, isoquercetin, and quercetin was higher than that in the blank control sample. It indicated that those active compounds were specifically adsorbed by the immobilized α-glucosidase. As it’s reported in the published works [26, 28], quercetin-3-*O*-glucuronide, isoquercetin, and quercetin have the ability to inhibit α-glucosidase. Thus, it was confirmed that active compounds in mulberry leaves included quercetin-3-*O*-glucuronide, isoquercetin, and quercetin.

**Table 2:** The data of compounds in lotus leaves obtained using HREIMS (A_1_: the peak area of the positive group; A_2_: the peak area of the blank control)

Peak no.	t_R_/min	Identification	Molecular formula	[M − H]^−^			A_1_/A_2_ (%)
				Detected	Expected	Error/ppm	
1	23.236	Quercetin-3-*O*-glucuronide	C_21_H_18_O_13_	477.0663	477.0670	− 1.5	132
2	24.017	Isoquercitrin	C_21_H_20_O_12_	463.0876	463.0877	− 0.2	109
3	39.950	Quercetin	C_15_H_10_O_7_	301.0348	301.0349	− 0.3	105
4	39.372	Kaempferol	C_15_H_10_O_6_	285.0404	285.0399	1.8	97

## Discussion

The paper microarray was prepared by polycaprolactone–chitosan-modification. Polycaprolactone was selected to modify filter paper for its hydrophobicity. Furthermore, its biodegradability and low melting point were also advantages for paper microarray preparation. For the purpose of convenient cutting and preparation, the pattern of paper microarray was designed to be rectangular as seen in Fig. 2c. The polycaprolactone-modified part constitutes the hydrophobic regions. It was prepared to restrict liquid in hydrophilic regions. In addition, it was designed to make it easy to grasp the paper microarray. Polycaprolactone would shrink when it cooled down after heating. Thus, “tortoise shell lines” were designed to avoid deformation of the modified paper.

By comparison between mass spectrometry chromatogram of the positive group and the blank control, it showed that immobilized α-glucosidase can specifically adsorb active compounds from the mulberry leaf extract and the lotus leaf extract. If the A_1_/A_2_ values of some compounds were close to 100%, it would be difficult to judge whether the compounds were specifically adsorbed or not. These compounds are suggested to be considered as potential bioactive compounds. After further verification experiments or literature survey, they may be verified as bioactive compounds. There were also some peaks with A_1_/A_2_ values much less than 100%, such as astragalin and quercetin. In Fig. 5c, the peak area is relatively large for these compounds. It means that they were non-specifically adsorbed by chitosan. The phenolic hydroxyl groups of astragalin and quercetin may react with the amino groups of chitosan.

After the immobilization of α-glucosidase, it is speculated that some of the adsorption sites were covered by enzyme, which led to the decrease in non-specific adsorption. For the compounds with obviously non-specific adsorption on the paper device, it is difficult to know the adsorption amount on α-glucosidase. Therefore, it is better to consider the compounds with obviously non-specific adsorption as potential active compounds. For the mulberry extract, Tao et al. [29] reported that isoquercitrin and astragalin can inhibit α-glucosidase activity. Wang et al. [30] reported that quercetin can inhibit α-glucosidase activity. They are all α-glucosidase inhibitors and should be considered as active compounds in mulberry leave extract.

In addition, some other methods can be attempted to decrease non-specific adsorption. For example, sodium periodate was reported to be a paper modifier [31]. Hydroxyl groups on cellulose will be oxidized to aldehyde groups by sodium periodate. Aldehyde groups can be used to immobilize enzymes. Unreacted sodium periodate can be washed easily. Therefore, non-specific adsorption will be decreased without using chitosan.

## Conclusion

An effective omics technology of TCM based on paper microarrays was reported in this work. An enzyme immobilized paper microarray was designed and fabricated in this work. The filter paper was modified by polycaprolactone which was 3D-printed on the paper. Then α-glucosidase immobilized paper microarrays were prepared successfully through cutting, chitosan modification, and enzyme immobilization. The colorimetric reaction results verifying the activity of immobilized α-glucosidase were retained. A cellphone was used to measure colorimetric reaction results. The paper microarray was used to discover active compounds in the mulberry leaf and the lotus leaf extracts. Through HREIMS analysis, active compounds were found being specifically adsorbed. The active compounds included chlorogenic acid in the mulberry leaf extract, and quercetin-3-*O*-glucuronide, isoquercetin, and quercetin in the lotus leaf extract. Some compounds with remarkable non-specific adsorption, such as astragalin and quercetin were also found to be active compounds. All of these compounds should be considered in the quality control of anti-diabetic drugs that are made from mulberry leaves and lotus leaves.

## Additional files

**Additional file 1.** Minimum standards of reporting checklist.

**Additional file 2.** The fixed parameters in the experiments for optimization of enzyme immobilization.

## References

[1] Kiyama R (2017). DNA microarray-based screening and characterization of traditional Chinese medicine. Microarrays.

[2] Zhang Q, Yang M (2010). DNA microarray technology and traditional Chinese medicines. Prog Nutr.

[3] Zhang YB, Wang J, Wang ZT, But PPH, Shaw PC (2003). DNA microarray for identification of the herb of dendrobium species from Chinese medicinal formulations. Planta Med.

[4] Wang LL, Li Z, Shao Q, Li X, Ai N, Zhao XP (2014). Dissecting active ingredients of Chinese medicine by content-weighted ingredient-target network. Mol BioSyst.

[5] Tao Y (2014). Studies on chemical biology approaches for rapid discovery of bioactive compounds from traditional Chinese medicine.

[6] Liu CX, Chen SL, Xiao XH, Zhang TJ, Hou WB, Liao ML (2016). A new concept on quality marker of Chinese materia medica: quality control for Chinese medicinal products. Chinese Tradit Herbal Drugs..

[7] Lourenço VK, Jiang ZJ, Zhang XQ, Curcino Vieira LC, Gonçalvez Corrêa A, Lucia Cardoso C (2013). Acetylcholinesterase immobilized capillary reactors coupled to protein coated magnetic beads: a new tool for plant extract ligand screening. Talanta.

[8] Wiekhorst F, Steinhoff U, Eberbeck D, Trahms L (2012). Magnetorelaxometry assisting biomedical applications of magnetic nanoparticles. Pharm Res.

[9] Wubshet SG, Brighente IMC, Moaddel R, Staerk D (2015). Magnetic ligand fishing as a targeting tool for HPLC-HRMS-SPE-NMR: α-glucosidase inhibitory ligands and alkylresorcinol glycosides from Eugenia catharinae. J Nat Prod.

[10] Zhuo RJ, Liu H, Liu NN, Wang Y (2016). Ligand fishing: a remarkable strategy for discovering bioactive compounds from complex mixture of natural products. Molecules..

[11] Liu LL, Ma YJ, Chen XQ, Xiong X, Shi SY (2012). Screening and identification of BSA bound ligands from *Puerariae lobata* flower by BSA functionalized Fe3O4 magnetic nanoparticles coupled with HPLC–MS/MS. J Chromatogr B.

[12] Deng X, Shi SY, Li SM, Yang TL (2014). Magnetic ligand fishing combination with high-performance liquid chromatography-diode array detector-mass spectrometry to screen and characterize cyclooxygenase-2 inhibitors from green tea. J Chromatogr B.

[13] Qing LS, Xue Y, Zheng Y, Xiong J, Liao X, Ding LS (2010). Ligand fishing from *Dioscorea nipponica* extract using human serum albumin functionalized magnetic nanoparticles. J Chromatogr A.

[14] Martinez AW, Phillips ST, Butte MJ, Whitesides GM (2007). Patterned paper as a platform for inexpensive, low-volume, portable bioassays. Angew Chem Int Ed.

[15] He Y, Wu WB, Fu JZ (2015). Rapid fabrication of paper-based microfluidic analytical devices with desktop stereolithography 3D printer. RSC Adv.

[16] Ahmed S, Bui M-PN, Abbas A (2016). Paper-based chemical and biological sensors: engineering aspects. Biosens Bioelect..

[17] Gu Z, Zhao MX, Sheng YW, Bentolila LA, Tang Y (2011). Detection of mercury ion by infrared fluorescent protein and its hydrogel-based paper assay. Anal Chem.

[18] Mitchell HT, Noxon IC, Chaplan CA, Carlton SJ, Liu CH, Ganaja KA (2015). Reagent pencils: a new technique for solvent-free deposition of reagents onto paper-based microfluidic devices. Lab Chip.

[19] Kaewarsa P, Laiwattanapaisal W, Palasuwan A, Palasuwan D (2017). A new paper-based analytical device for detection of glucose-6-phosphate dehydrogenase deficiency. Talanta.

[20] Chen JG, Bu WL, Lai WQ, Liu DY, Mei S, Liu Z (2011). Hypoglycemic effects and mechanism of mulberry leaves polysaccharide. Chin Tradit Herbal Drugs..

[21] Zhao J, Gao L, Qi ZP (2005). Extraction of folium nelumbinis total alkaloid and its salt and comparison in lowering blood lipid between them. Tianjin J Tradit Chin Med.

[22] Sheldon RA (2007). Enzyme immobilization: the quest for optimum performance. Adv Synth Catal.

[23] Nery EW, Kubota LT (2016). Evaluation of enzyme immobilization methods for paper-based devices—a glucose oxidase study. J Pharm Biomed Anal.

[24] Guo SX, Shao JY, Gong XC (2018). Paper-based analytical devices prepared with polycaprolactone printing and their application in the activity determination of mulberry extracts. J Pharm Biomed Anal.

[25] Zhao TT, Wei H, Chen LM, Wang ZM, Zhang QW, Zhao ZB (2017). HPLC fingerprints of different medicinal parts of morus alba L. Chin Pharm J.

[26] Cheng YY, Wang Y. The compounds with inhibitory activity on α-glucosidase in lotus leaves and its use. China, CN 102827221 A. 2012.

[27] Liu XH, Li ML, Tan B, Chen HH, Lu Y (2014). Inhibitory effects of chlorogenic acid and isochlorogenic acid from purple sweet potato leaves on α-glucosidase. Modern Food Sci Technol.

[28] Cheng JY (2013). Screening study on the α-glucosidase inhibitors from diospyros kaki leaves.

[29] Tao Y, Zhang YF, Cheng YY, Wang Y (2013). Rapid screening and identification of α-glucosidase inhibitors from mulberry leaves using enzyme-immobilized magnetic beads coupled with HPLC/MS and NMR. Biomed Chromatogr.

[30] Wang SH, Huang WL, Chen QS, Zeng L, Zeng FJ (2012). Inhibition of rutin and quercetin on α-glycosidase. China Brewing..

[31] Mu C, Guo J, Li X, Lin W, Li D (2012). Preparation and properties of dialdehyde carboxymethyl cellulose crosslinked gelatin edible films. Food Hydrocolloids.

