# Supplementary material for: Fabrication of paper-based enzyme immobilized microarray by 3D-printing technique for screening α-glucosidase inhibitors in mulberry leaves and lotus leaves
Source: Chin Med. 2019 Mar 29;14:13. doi: 10.1186/s13020-019-0236-y (PMC6440092; doi:10.1186/s13020-019-0236-y)
Supplement: Supplementary file 2 — Additional file 2. The fixed parameters in the experiments for optimization of enzyme immobilization. [file 13020_2019_236_MOESM2_ESM.docx]

**Fabrication of paper-based enzyme immobilized microarray by 3D-printing technique for screening α-glucosidase inhibitors in mulberry leaves and lotus leaves**

Shangxin Guo†, Xiaotong Lin†, Yi Wang, and Xingchu Gong*

Pharmaceutical Informatics Institute, College of Pharmaceutical Sciences, Zhejiang University, Hangzhou 310058, China

* Correspondence: gongxingchu@zju.edu.cn; Tel.: +0086-571-88208426; Fax: 0086-571-88208426

† These authors contributed equally to this work.

**Additional Information**

**Table S1.** The fixed parameters in the experiments observing the influences of Na_2_CO_3_ solution concentration and volume

| Parameter | Value |
| --- | --- |
| Chitosan solution volume | 100μL |
| Glutaraldehyde concentration | 0.18mol/L |
| Time of glutaraldehyde crosslinking | 2h |
| Time of α-glucosidase immobilization | 3h |

**Table S2.** The fixed parameters in the experiments observing the influences of glutaraldehyde concentration

| Parameter | Value |
| --- | --- |
| Chitosan solution volume | 100μL |
| Na_2_CO_3_ solution concentration | 5mmol/L |
| Na_2_CO_3_ solution volume | 50μL |
| Time of glutaraldehyde crosslinking | 2h |
| Time of α-glucosidase immobilization | 3h |

**Table S3.** The fixed parameters in the experiments observing the influences of time of glutaraldehyde crosslinking

| Parameter | Value |
| --- | --- |
| Chitosan solution volume | 100μL |
| Na_2_CO_3_ solution concentration | 5mmol/L |
| Na_2_CO_3_ solution volume | 50μL |
| Glutaraldehyde concentration | 0.18mol/L |
| Time of α-glucosidase immobilization | 3h |

**Table S4.** The fixed parameters in the experiments observing the influences of time of α-glucosidase immobilization

| Parameter | Value |
| --- | --- |
| Chitosan solution volume | 100μL |
| Na_2_CO_3_ solution concentration | 5mmol/L |
| Na_2_CO_3_ solution volume | 50μL |
| Glutaraldehyde concentration | 0.18mol/L |
| Time of glutaraldehyde crosslinking | 2h |
